# Effect of a theory-based nutrition education intervention during pregnancy through male partner involvement on newborns’ birth weights in Southwest Ethiopia. *A three-arm community based Quasi-Experimental study*

**DOI:** 10.1371/journal.pone.0280545

**Published:** 2023-01-17

**Authors:** Dereje Tsegaye, Dessalegn Tamiru, Tefera Belachew

**Affiliations:** Department of Nutrition and Dietetics, Institute of Health, Jimma University, Jimma, Ethiopia; University of Chicago, UNITED STATES

## Abstract

**Background:**

Low birth weight is one of the most serious public health issues affecting newborns, with estimates ranging from 15% to 20% of all births worldwide. According to the Ethiopian demographic health survey report, the prevalence of Low Birth Weight rose from 11% in 2011 to 13% in 2016. The high proportion of birth weight in Ethiopia is hypothesized to be due to inadequate maternal diet which is associated with poor nutrition education during pregnancy. This study aimed to assess the effect of theory-based nutrition education during pregnancy through male partner involvement on birth weight in rural parts of the southwest Ethiopia.

**Study design:**

A community-based quasi-experimental study was conducted.

**Methods:**

A total of 403 pregnant women were selected from 22 rural kebeles of Illu Aba Bor Zone, Southwest Ethiopia from June to December 2019. Participants were assigned to one of the three study arms: Couple group:—husband and wife received nutrition education together, women alone:—pregnant women received the nutrition education alone and control group:—received the routine care during Antenatal care. The nutrition education was guided by theory of planned behavior. Monthly home visits were made to the pregnant women in the intervention groups and leaflets with key counseling messages were distributed to each woman in the intervention arms. A structured interviewer-administered questionnaire was used to collect the data. A qualitative 24‐h dietary recall was used to assess dietary data, and the Mid‐Upper Arm Circumference was used to assess nutritional status. Birth weight was measured within 24 hours of birth. Analysis of variance, linear mixed-effects model, and mediation analysis were used to assess effect of the intervention on birth weight.

**Results:**

A higher proportion of the newborns in the control group had low birth weight as compared to the couple group and the women alone group (18.1% vs 7.0% vs 11.5%, *p = 0*.*037*) respectively. The mean birth weight of babies born to women from the couple group was 0.42 kg greater than that of newborns born to women in the comparison group (3.34 vs 2.92 kg, *p< 0*.*001*). The linear mixed effect model showed that the average birth weight of babies born from women in the couple group was 0.40 kg higher than that of the control group (β = 0.400, *P<0*.*001*). The direct effect of the intervention on birth weight of babies born from women in the couple group was 0.23 (β = 0.227, *P<0*.*001*) whereas the indirect effect mediated by maternal dietary diversity practice was 0.18 (β = 0.178, *P<0*.*001*), accounting for 43.9% of the total effect of the intervention.

**Conclusion:**

The involvement of males and the application of the theory of planned behavior in nutrition education interventions during pregnancy resulted in improved birth weight. Maternal dietary diversity mediated the effect of nutrition education on birth weight. The findings highlight the implication of improving pregnant women’s nutrition education through male involvement and the application of theories to improve birth weight.

## Background

The World Health Organization (WHO) defines low birth weight (LBW) as a birth weight of less than 2500 grams regardless of gestational age [1]. It is the result of either intrauterine growth restriction or premature birth. Globally, it is estimated that 15% to 20% of all births are of low birth weight [2]. Regional estimates show that the rates of low birth weight are 28% in South Asia, 13% in sub-Saharan Africa, and 9% in Latin America. According to a systematic review and meta-analysis, the pooled prevalence of LBW in Ethiopia was 17.3% and the subgroup analysis revealed the prevalence rate of LBW was 22% in Addis Ababa, 18.1% in Tigray, 16.8% in Oromia, 16% in Amhara, and 1.3% in South Nations and Nationalities People’s region (SNNPR) [3]. The Ethiopian demographic health survey (EDHS) report showed an increasing trend in the prevalence of LBW from 11% in 2011 to 13% in 2016 [4]. Local surveys showed that the prevalence of LBW ranged from 7.8% to 18% [5, 6]. Different descriptive and analytical studies show the common risk factors for low birth weight in Ethiopia are preventable causes [7].

Worldwide, LBW is one of the major public health problems that put forth short-term health effects, like hypoglycemia [8], and hypothermia [9], and increases the risk of non-communicable diseases later in life [10]. Low birth weight increases the risk of death by threefold, and the risk further increases as birth weight decreases. It indirectly accounts for 60% of neonatal deaths and more than 13 million disability-adjusted life years among children under the age of five [11]. According to a study in low- and middle-income countries, LBW is associated with a 2.5- to 3.5-fold increased risk of wasting, stunting, and underweight in children [12].

LBW is a key indicator of the progress towards the achievement of the global nutrition targets [13], as a 30% reduction in the number of LBW live births between 2012 and 2025 is one of the six global nutrition targets [14]. The global nutrition targets require an average annual rate of reduction of 2.74% per year. However, the average annual rate of reduction is 1.23% per year, which shows the progress toward reducing the prevalence of LBW is not satisfactory [15].

Nutrition education is an essential consideration for improving the health of women of reproductive age and pregnancy outcomes. Nutrition education programs are important as they target enhancing dietary intake by promoting behavioral changes such as food choice and cooking ability, goal-setting, motivation, and supporting efforts for change. Dean et al., for example, discussed how preconception nutrition-specific interventions, specifically increased folic acid, and multivitamin supplements, resulted in positive pregnancy outcomes in pregnant women [16]. Furthermore, Rao demonstrated an improvement in hemoglobin level through a nutrition awareness program comprising informal meeting sessions, cookery activities to disseminate knowledge about the use of iron-rich foods, and kitchen garden activities [17].

Theory and models can be used to identify individual characteristics and the surrounding environment, which can aid in behavior change as a result of educational interventions. Behavior modification theories and models can be extremely beneficial in the development and evaluation of comprehensive educational programs [18]. The Theory of Planned Behavior (TPB) is a popular behavior change theory in nutrition education. According to this theory, three determinants can predict an individual’s intention: attitude toward the behavior, beliefs about motivation to comply with other expectations, and beliefs about the perceived level of control over factors that can either facilitate or hinder behavior performance [19]. This theory is a suitable framework to use during pregnancy because it includes factors that may be influenced by pregnancy-related factors [20].

**Fig 1:**
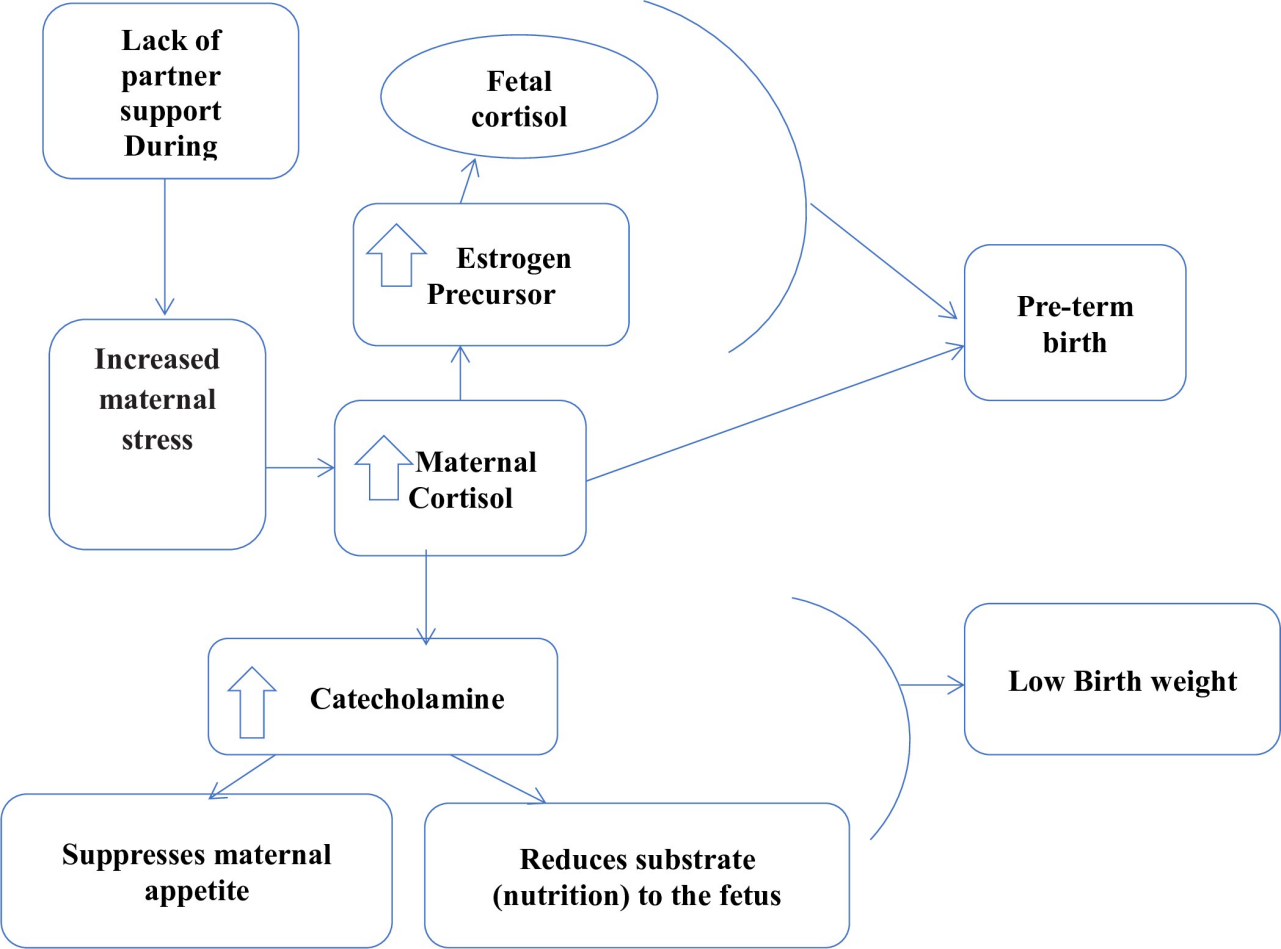
Mechanisms and pathways of male involvement to birth outcome adapted from literature [28].

Involving husbands in nutrition-related social behavior-change programs is particularly crucial in countries where women’s decision-making autonomy is limited and households are typically headed by men [21]. Recent evidence also highlights the need to consider the broader social, cultural and economic factors, including the value of involving men in childcare, when designing nutritional interventions [22]. Male partner involvement during pregnancy can promote positive maternal behaviors like healthier eating, earlier attendance and adherence to antenatal and postnatal health care [23]; and reduce risks of preterm birth, low birth weight, fetal growth restriction, and infant mortality [23–26]. There is epidemiological and physiological evidence that male involvement (emotional, logistical and financial support) reduces maternal stress [24, 27]. A woman may experiences stress due to the lack of a male partner’s involvement and accessibility, which raises the level of maternal cortisol released during pregnancy. As a result, there is an increased release of catecholamine that will either alter the mother’s appetite or decrease the amount of substrate (nutrition) passing to the fetus. Consequently, preterm birth rates and low birth weight rates will rise. The absence of a male partner’s support during pregnancy, on the other hand, will induce the estrogen precursor to be stimulated, which will then have an impact on the fetal stress response and the production of fetal cortisol, which will ultimately have an impact on the birth outcome [28]. The Fig 1 below describes the mechanism and pathways of biologic process inside the body of women experiencing stress during pregnancy.

Male involvement interventions in maternal health [29] and reproductive health, particularly in the prevention of mother-to-child transmission (PMTCT) of HIV [30], have demonstrated significant positive effects in areas such as postnatal consultation attendance, PMTCT regimen uptake, and adherence. However, However, the effects of male engagement on nutrition- or child-health-related behaviors and outcomes, have not been thoroughly studied [31]. Hence, the objective of this study was to assess the effects of nutrition education intervention through male partner involvement on the birth weight of newborns in Ilu Aba Bor Zone.

## Methods

### Study setting and period

A community-based quasi-experimental study was conducted among pregnant women in rural communities of Ilu Aba Bor zone, Southwest Ethiopia. The zone is one of the 21 zones of Oromia National Regional State, located at a distance of 600 km from the capital in the southwest direction. It is located in the western part of the region, between 340 52 12 "E and 410 34 55E longitude and 70 27 40N and 90 02N latitude. The zone is divided into 14 districts, each with 23 urban and 263 rural kebeles and a population of 934,783, with 467,553 males and 465,792 females. Agriculture is the dominant means of livelihood in the Zone. The study was conducted from July to December 2019.

### Study population and sampling technique

Pregnant women in their first and early second trimesters (up to 16 weeks of gestation) were the study participants. G*Power 3.0.10 [32] was used to calculate the sample size. The following assumptions were used to calculate the required sample size: a 95% confidence level, a 5% margin of error, an 80% power, and a 0.25 effect size (hypothetical difference in birth weight between the intervention and control groups). A design effect of 2 was used, and a 10% loss to follow-up was taken into account. The total sample size calculated was 350. However, the sample size calculated for the baseline survey as part of this project [33] was larger, so that was considered.

### Inclusion and exclusion criteria

We enrolled all consenting married pregnant women in their first 16 weeks of gestation because the intervention was to be implemented before the delivery period. Furthermore, pregnant women who had lived in the study area for at least 6 months were included to maintain homogeneity in access to nutrition-related information and health services. Pregnant women with the diagnosis of Hypertension, Diabetes Mellitus requiring a special diet and nutritional needs were excluded. Participants were also not included in the analysis for others reasons such as abortion, failure to comply, relocation, and not informed birth weight.

### Allocation to the study arm

**Fig 2:**
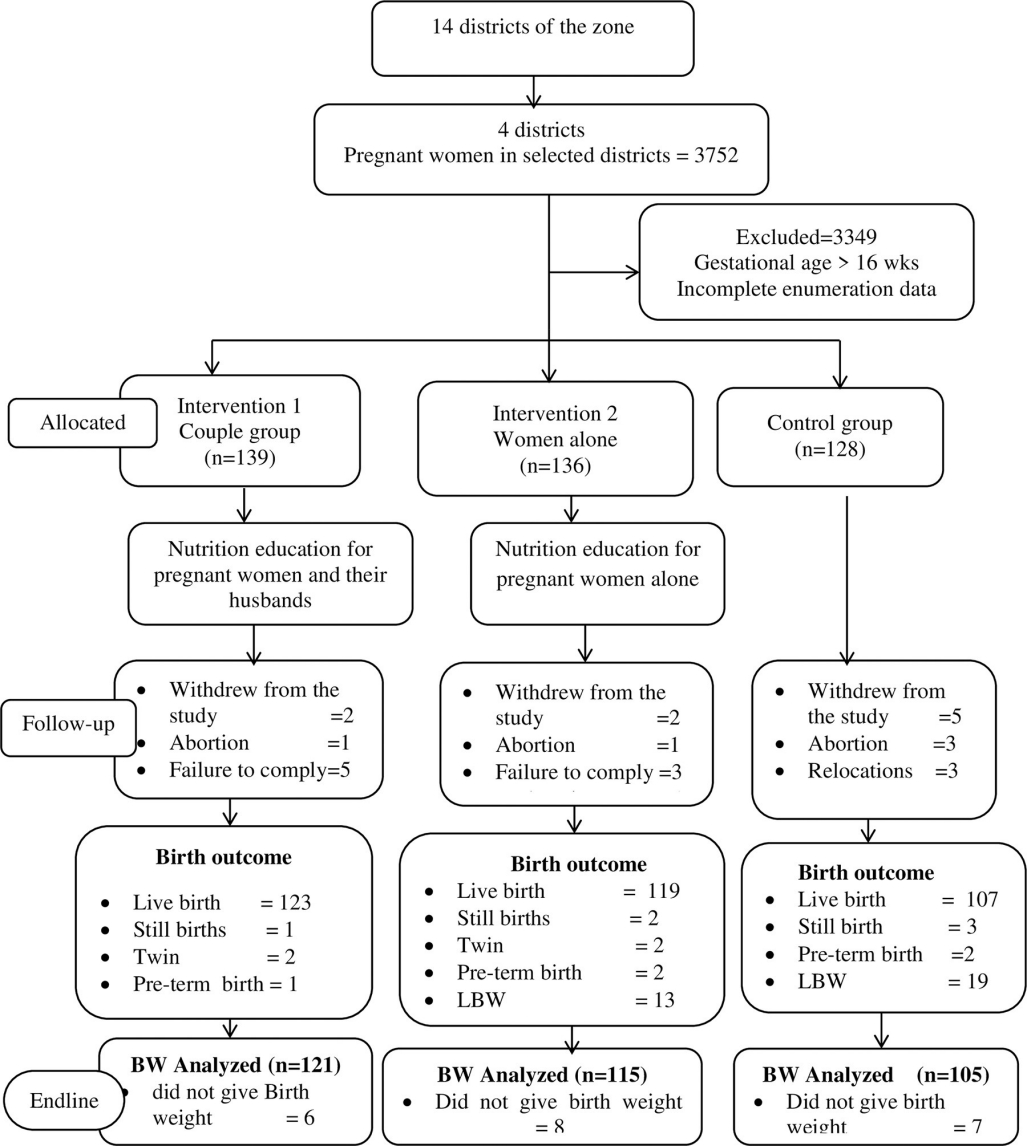
Trial flow diagram of the progress of study participants through the phases of the intervention. Note: live birth includes the twins and preterm births BW–birth weight.

In the first stage, four districts were selected. In the second stage, these districts were stratified into three according to the geographic direction and proximity to each other. The districts were allocated to a control group or one of the two intervention groups. Then, Kebeles (the lowest administrative unit) were selected at random from the districts that were selected. Pregnant women were enumerated using house-to-house visits in all of the selected Kebeles, and all that fulfilled the inclusion criteria were included in the study (Fig 2). Due to the nature of the intervention, the participants and intervention implementers were not blinded to the allocation.

### Intervention

A theory-guided community-based nutrition education intervention on maternal diets was used. The current study was not intended to test a theoretical model; rather, it was useful in directing what types of variables and processes might be important in shaping maternal health behaviors and thus required attention in the intervention. Participants were assigned to one of the three study arms: Couple group:—husband and wife received health education together, women alone:—pregnant women received health education alone and control group:—received no education.

After reviewing the World Health Organization’s (WHO) guidelines and the Training of Trainers Manual of the Federal Democratic Republic of Ethiopia, the nutrition education protocol was established [34, 35]. The education protocol’s main points were to increase the amount and duration of meals as the pregnancy progresses, as well as to diversify the meals. During each education session, the importance of taking an iron/folic acid supplement and using iodized salt was also stressed. Furthermore, the education protocol included reducing the workload, getting enough rest each day, and using maternal health care facilities. The benefits of consuming a balanced diet and an adequate amount of food were included in the counseling protocol. The consequences of taking insufficient nutrients were also deliberated during the education session. The intervention protocol was described in a previously published paper [33].

Two approaches were used to implement the intervention. These include group nutrition education for intervention groups as well as monthly home visits and counseling for pregnant women to assist them in adopting the recommended practices after the nutrition education. Group nutrition education was given monthly at health posts to husbands and pregnant women together in one group and women alone in the other. The nutrition education was given monthly for three sessions and each education session lasted for 45 to 60 minutes. In addition, one health extension worker (HEW) was assigned to each intervention village to provide counseling and support to mothers. Before going into the field, the HEWs were trained and given counseling resources.

All pregnant women seeking antenatal care (ANC) received nutrition counseling following a national standard protocol. Pregnant women in both the control and intervention groups had access to this service.

### Fidelity check

The intervention’s fidelity was assessed using best practice recommendations developed by the National Institutes of Health (NIH) Behavioral Change Consortium. The criteria were used to evaluate the intervention design, counselor training, counseling process, intervention receipt, and enactment of skills gained from the intervention [36]. The intervention fidelity is described in detail elsewhere [33].

### Data collection tools and procedures

A pre-tested structured interviewer-administered questionnaire was used to collect data on dietary diversity practice and demographic characteristics, such as household wealth status, household food security status, obstetric-related factors, and the intervention. The tool was developed after a review of various literature and standard guidelines [4, 37–39] The tool was first written in English, and translated into the local language (Afan Oromo), and then translated back to English to ensure consistency. Eight BSc nurses collected the data, supervised by four masters of Public Health professionals and the principal investigator. The data collectors and supervisors received five days of training on the data collection tools and procedures.

The primary and secondary outcomes of this intervention were the dietary diversity and nutritional status of the pregnant women, respectively. The final outcome, birth weight was assessed using a digital weighing scale (SECA 876, Hannover, Germany) and recorded by the midwives within 24 hours of the baby’s birth. Before each measurement, the scale was calibrated with a known-weight object. Furthermore, before weighing each newborn, the reading on the scale was reset to zero. The same professionals determined stillbirth (no indications of life at birth after 24 weeks of gestation) and pre-term birth (PTB) (before 37 weeks of gestation) at birth.

The dietary diversity of individual women was determined using a 24-hour qualitative dietary recall. Women’s dietary diversity (WDD) is a nine-food-group score that is used to assess a diet’s micronutrient sufficient [37]. The pregnant women were asked to recall what they had eaten and drank in the previous 24 hours, both inside and outside their homes. Mid Upper Arm Circumference (MUAC) was used to assess nutritional status of the pregnant women. The procedure involved measuring the distance from the acromion to olecranon processes while the respondents’ elbow was flexed to 90 degree. The midpoint was marked, and measuring tape was placed snugly around the arm at the midpoint mark while hanging arm freely.

The Ethiopian Demographic and Health Survey’s wealth constructs were used to assess household wealth status, which included household assets, utilities, and housing features [4].

### Data processing and analysis

The collected data were entered into Epi data version 3.5.1 and then exported to SPSS version 23 for analysis. Summary statistics such as mean and percentages were used to describe the study population based on the study outcomes, demographic features, and other pertinent risk factors. The analysis did not include multiple births, premature births, and stillbirths. A chi-squared test was used to compare the baseline characteristics of the two interventions and control groups. Analysis of Variance (ANOVA) was used to compare means between the control and intervention groups, and when ANOVA was statistically significant, a post hoc test (Tukey HSD test) was used to determine the level of significance of values between and within groups. A p-value of less than 0.05 was considered statistically significant.

The food groups were categorized into nine food groups. Finally, the food groups were summed to generate a dietary diversity score (DDS), which was ranked into tertile. A detailed description of the dietary assessment is described elsewhere [33]. Principal component analysis was used to construct the wealth index. Then the wealth index was classified into wealth quintiles. The procedure of the wealth index is described elsewhere [40].

The linear mixed-effects model was used to examine the effect of the intervention on birth weight considering kebeles as clusters and birth weights nested within the clusters. Kebeles were analyzed as random effects due to the correlation of observations due the clustering of individuals within the selected Kebeles. The model was adjusted for potential confounders such as maternal age, maternal education, maternal occupation, family size, newborn sex, baseline MUAC, and baseline DDS. Before fitting the model, the normality assumption of the outcome variable, birth weight, was tested using Shapiro-Wilk’s test, and the test revealed that the assumption was satisfied (p > 0.05). We used the Akaike information criterion (AIC) to help us choose the best statistical model. We selected the model that had the smallest AIC. Variables in the bivariate linear mixed regression model with p-values less than 0.2 were selected as candidate variables for the multivariable linear mixed model analysis. The effectiveness of the intervention was determined by examining the interaction between time and the intervention.

Furthermore, the nutrition education intervention provided to pregnant women on the importance of eating a diverse diet may have both direct and indirect effects on birth weight. Taking this into account, mediation analysis was also performed to assess the intervention’s direct and indirect effects on birth weight. Birth weight was the outcome variable and intervention was the predictor of the outcome. The dietary practice was the mediator of the intervention effect. The PROCESS macro version 3.4 for SPSS, developed by Andrew F Hayes [41], was used to implement the regression-based path analysis. To explore the relationship between variables and birth weight, a linear regression model was fitted before path analysis. Then, using mediation, the effect of the nutrition education intervention on birth weight was evaluated. The bootstrapping method was employed to determine the magnitude of the mediation effect (made for 5000 drawings). This technique estimates the indirect effect (generating an empirical representation of the sample distribution, treated as a population representation). Participants were also not included in the analysis for others reasons such as abortion, failure to comply, relocation, and not informed birth weight.

### Ethics approval and consent to participate

The study followed the Helsinki Declaration on Human Subjects Medical Research [42]. The Institutional Review Board (IRB) of Jimma University Institute of Health reviewed the protocol and provided ethical clearance (Ref. No. IHRPGD/595/2019). The local authorities were informed about the research through an official letter from the university to obtain their permission. Written informed consent was obtained from the participants after clear and adequate information was provided to them about the research and their right to participation, including their right to decline participation any time they feel to do so. During the study follow-up, mothers and newborns that had health problems were referred to a nearby health facility to seek proper medical care. Mothers with low birth weight babies were counseled on proper newborn care, such as feeding, proper cleanliness, and sanitation, to prevent mortality in this very vulnerable population.

## Results

### Socio-demographic characteristics of pregnant women

A total of 341 newborns (couple group = 121, women alone = 115, and control = 105) birth weight was measured within 24 hours of birth and included in the final analysis. The socio-demographic characteristics of all three groups were comparable at baseline (Table 1). The flow of the study participants through the trial process is depicted in (Fig 2).

**Table 1:** Baseline socio-demographic and economic characteristics of the pregnant women in Illu Aba Bor zone, Southwest Ethiopia, 2019.

Characteristics	Category	Couples (n = 121) N (%)	Women alone(n = 115) N (%)	Control (n = 105) N (%)	*P*
**Age of the Mother**	≤ 24	61(50.4	70(60.9)	57(54.3)	0.495
	25–34	57(47.1)	41(33.0)	44(41.9)	
	≥ 35	3(2.5)	4(6.1)	4(3.8)	
**Religion**	Orthodox	26(21.5)	27(23.5)	21(20.0)	0.358
	Muslim	69(57.0)	66(57.4)	71(67.6)	
	Protestant	26(21.5)	22((19.1)	13(12.4)	
**Ethnicity**	Oromo	101(83.5)	102(88.7)	95(90.5)	0.491
	Amhara	17(14.0)	10(8.7)	9(8.6)	
	Others	3(2.5)	3(2.6)	1(0.9)	
**Educational status of the mother**	No formal education	40(33.1)	27(23.5)	27(26.7)	0.164
	Primary	59(48.8)	60(52.2)	63(57.1)	
	Secondary & above	22(18.2)	28(24.3)	15(16.2)	
**Educational status of the husband**	No formal education	28(23.1)	16(13.9)	19(18.1)	0.249
	Primary	72(59.5)	68(59.1)	60(57.1)	
	Secondary & above	21(17.4)	31(27.0)	26(24.8)	
**Occupational status of the Mother**	Employee	4(3.3)	5(4.3)	3(2.9)	0.733
	Merchant	9(7.0)	12(10.4)	6(5.7)	
	Housewife	103(85.0)	93(80.9)	94(89.5)	
	Daily laborer	5(4.1)	5(4.3)	2(1.9)	
**Family Size**	<5	92(76.0	99(86.1)	88(83.8)	0.110
	≥5	29(24.0)	16(13.9)	17(16.2)	
**Wealth Quintile**	Lowest	21(17.4)	24(20.9)	13(12.4)	0.112
	Second	36(29.8)	21(18.3)	20(19.0)	
	Middle	19(15.7)	25(21.7)	18(17.1)	
	Fourth	22(18.2)	27(23.5)	25(23.8)	
	Highest	23(19.0)	18(15.7)	29(27.6)	

Others include Gurage, Tigray

### Effect of nutrition education on dietary diversity and nutritional status of the pregnant women

According to the findings of this study, the baseline mean dietary diversity scores among the three groups were comparable (F = 2.644, *P = 0*.*072*). After nutrition education, there was a statistically significant difference in mean DDS among the three groups (F = 15.295, *P<0*.*001*) (Table 2). Similarly, the baseline nutritional status was comparable (F = 0.487, *P = 0*.*615*). However, there was a statistically significant difference in the end-line nutritional status among the three groups (F = 10.541, *P<0*.*001*) (Table 2). More women in the intervention group gave birth in a health facility than women in the control group (89.7% vs 84.9% vs 77.1%, *P = 0*.*0378*).

**Table 2:** Comparison of the Mean DDS and MUAC among the pregnant women ILU Aba Bor Zone, Southwest Ethiopia, 2019/20.

Variables	Couple group Mean(±SD)	Women alone Mean(±SD)	Control Mean(±SD)	*F-Test*	*p*
	
** DDS**					
** Baseline**	4.95±1.01	4.60±1.09	4.81±1.19	2.678	0.37
** End line**	6.82±1.04	6.29±1.49	5.88±1.13	17.48	<0.001
**MUAC**					
** Baseline**	22.90±1.57	22.72±1.49	22.73±1.59	0.023	0.615
** End line**	24.43±1.47	23.9±1.74	23.5±1.64	13.05	<0.001

***p<0.001 *p<0.05 SD–Standard deviation

### Effects of nutrition education on birth weight

A higher proportion of the newborns in the control group had low birth weight as compared to the couple group and the women alone group (18.1% vs 6.7% vs 11.3%, p = 0.028) respectively (Table 3). The mean (±SD) birth weight of the newborn in the couples’ group was 3.34 kg (±0.50), while it was 3.07 kgs (±0.47) among the women alone and 2.92 kgs (±0.47) in the control group. The mean birth weight difference between the couple and women alone as well as the couple and the control group was statistically significant (p < 0.001), while there was a marginally significant difference between the women alone and the control group (Table 4).

**Table 3:** Characteristics of the new born babies, Illu Aba Bor Zone, Southwest Ethiopia, 2019/20.

Variables	Category	Couple group N (%)	Women alone N (%)	Control N (%)	*p*
**Sex of the child**	Male	67(55.4)	62(53.9)	51(48.6)	0.533
	Female	54(44.6)	53(46.1)	54(51.4)	
**Birth weight**	Low(< 2500g)	8(6.7)	13(11.3)	19(18.1)	0.028
	Normal(2500 – 4000g)	113(93.3)	102(88.7)	86(81.9)	

**Table 4:** Comparison of the newborn babies’ mean birth weight among the intervention and control group, Illu Aba Bor, Zone, Southwest Ethiopia, 2019/20.

Variables	Couple group Mean(±SD) Kgs	Women alone Mean(±SD) Kgs	Control Mean(±SD)Kgs	Mean difference		
				Couple Vs control	Couple Vs Woman alone	Woman alone Vs control
**Birth weight**	3.34±0.52	3.07±0.47	2.92±0.47	0.41***	0.26***	0.14

***p<0.001 *p<0.05 SD–Standard deviation Kg—Kilogram

The average birth weight varied significantly between clusters (*p = 0*.*04*) in the first model (empty model). The intra-cluster correlation coefficient was 0.068. The average birth weight in the couple group was 0.40 kgs higher and in the women alone group 0.14 kgs higher than the control group (β = 0.400, *P < 0*.*001*), according to the linear mixed-effects model (Table 5).

**Table 5:** Linear Mixed-Effects Model predicting birth weight of newborns in Illu Aba Bor, Zone Southwest Ethiopia, 2019/20.

Fixed Effect	Model 1		*p*	Model 2		*p*	Model 3		*p*
Variables	Estimate	95% CI		Estimate	95% CI		Estimate	95% CI	
**Intercept**	3.11	3.01,3.22	<0.001	3.11	3.02,3.18	<0.001	3.09	2.85,3.33	<0.001
**Intervention effect**									
**Couple group**				0.40	0.22,0.59	<0.001	0.40	0.22,0.58	<0.001
**Women alone group**				0.13	-0.06,0.31	0.058	0.14	-0.04,0.29	0.047
**Random effect**									
**Level two variance**	0.22		0.04	0.22			0.21		
**AIC**	528.44			514.70			523.65		
**ICC**	0.068			0.064			0.061		

Note: Model 1. Intercept‐only model; Model 2:Slope‐only model; Model 3. Intercept with slope model

SE–Standard error DDS = Dietary Diversity Score MUAC–Mid upper circumference CI–Confidence interval

AIC–Akaike’s information criterion ICC—Intra-cluster correlation

The model was adjusted for maternal education, maternal age, maternal occupation, wealth status, family size, infant sex, baseline MUAC, and baseline DDS.

**Fig 3:**
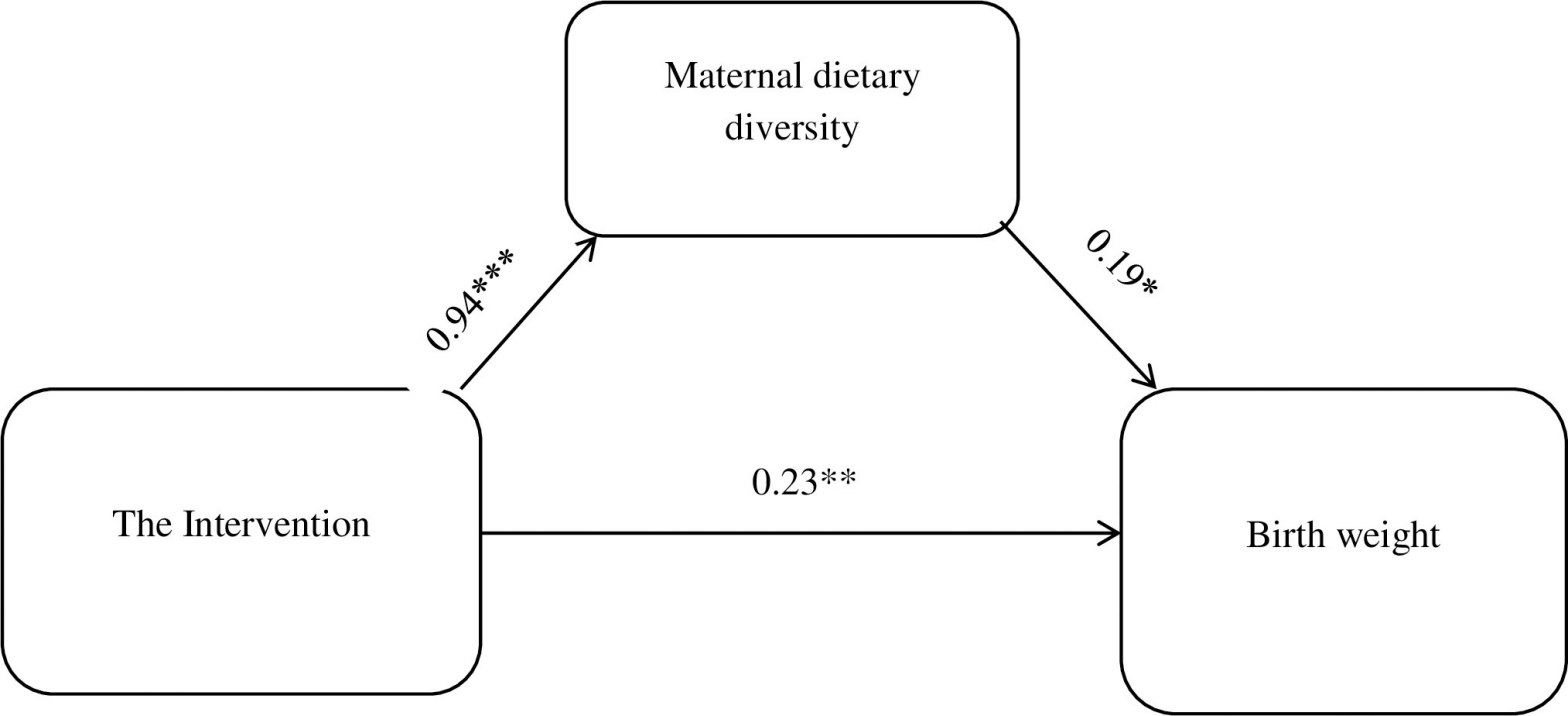
The indirect effect of nutrition education intervention through male involvement on birth weight, Ilu Aba Bor, zone southwest Ethiopia, 2019/20. Note: ***p<0.001, **p <0.01, *p<0.05.

According to the mediation analysis of linear models, the direct effect of nutrition education among the couple group on birth weight was 0.23 (β = 0.227, 95% CI = (0.123, 0.443). The estimate of indirect effect indicated that the effect of nutrition education through male involvement mediated through a change in maternal dietary diversity practice resulted in increased birth weight by 0.18 (β = 0.178, 95% CI (0.088, 0.251)). The total effect of the intervention, on average, increased birth weight by 0.40 (total effect). Maternal dietary diversity practice mediated 43.9% of the effect of the intervention on birth weight (Fig 3) **(**Table 6).

**Table 6:** Analysis of the direct and indirect effect of the intervention on the birth weight in Illu Aba Bor zone, Southwest Ethiopia,2019/20.

Variables	Estimate(SE)	95% CI
**The total effect of the intervention on birth weight**		
**Intervention 1 (Couple group)**	0.40(0.06)	0.28.0.53
**Intervention 2 (Women alone group)**	0.14(0.06)	0.02,0.27
**A direct effect of the intervention on birth weight**		
**Intervention 1 (Couple group)**	0.23(0.07)	0.12,0.44
**Intervention 2 (Women alone group)**	0.10(0.06)	0.02,0.26
**The indirect effect of the intervention on birth weight**		
**Intervention 1 (Couple group)**	0.18(0.02)	0.09,0.25
**Intervention 2 (Women alone group)**	0.04(0.01)	0.03,0.09
**Effect of intervention on dietary diversity practice**		
**Intervention 1 (Couple group)**	0.94(0.94)	0.66,1.22
**Intervention 2 (Women alone group)**	0.21(0.17)	0.07,0.74
**Effect of dietary diversity practice on birth weight**	0.19(0.02)	0.09,0.28

The model was adjusted for maternal education, maternal age, maternal occupation, wealth status, family size, infant sex, baseline MUAC, and DDS.

DDS–Dietary diversity score MUAC–Mid Upper Arm circumference SE–Standard error

## Discussion

The aim of the study was to assess the effect of theory-based nutrition education intervention through male involvement on birth weight. After adjusting for potential confounders, the linear mixed-effects model revealed a higher birth weight in the couple’s group compared with the women alone and the control group. Accordingly, the mean birth weight of babies born to women who attended nutrition education with their husbands was 0.40 kg higher than that of babies born to women in the control group and 0.26 kg higher than that of babies born to women who attended nutrition education alone. The difference could be attributed to the difference in the intervention approaches. Pregnant women in the couple group participated in nutrition education with their husbands. The male partner’s participation in the nutrition education may have enhanced their emotional, logistical, and financial support to the pregnant women, which may have reduced the stress burden, improved maternal behaviors (dietary practices, care seeking, lifestyle) and enhanced maternal wellbeing and delivery outcomes. The women alone group received the nutrition education alone, whereas the control group received routine education during antenatal care. A similar finding was reported in a study in Nepal, in which women who were educated alongside their husbands were more likely than women who received education alone to attend a postpartum visit [43]. Another study also revealed the effect of family meetings on the completeness of care, which was strongest when women attended with a family member [44]. This could be due to increased health-related communication and engagement between couples during or after the education sessions, resulting in a better understanding and/or retention of new information. It is also because of increased support from the family and their spouses due to the better understanding of the importance of nutrition during pregnancy.

The intervention had a direct effect of 0.23 on birth weight. One explanation for the intervention’s immediate positive effect is the fundamental information provided in the nutrition education, which may have helped women better understand what makes a healthy diet during pregnancy, which may have further improved healthy behaviors and led to an increase in birth weight. A similar finding was reported in a study in the West Gojam Zone of Ethiopia in which theory-based nutrition education had a direct effect on birth weight [45].

The intervention effect on birth weight was mediated by women’s dietary diversity practices. This could be because women in the couple group received nutrition education about maternal diet using the TPB, which may have increased women’s knowledge of the consequences of poor diet, the benefits of eating a balanced diet, food preparation, and weight gain, which in turn improved birth weight. Further, the male partner involvement in the nutrition education during pregnancy may have promoted positive maternal behaviors such as healthier eating that may have reduced risk of fetal growth restriction and low birth weight. Furthermore, nutrition education may have changed the cultural beliefs that tended to limit the consumption of a diversified diet. This result is in line with a systematic review of studies on the effect of nutrition interventions on birth weight, which found that nutrition interventions had an indirect effect on birth weight [46].

The findings have practical implications for preventing low birth weight. The fact that significantly lower numbers of newborns have LBW in the couple group implies the need for enacting community-level education through male partner involvement using theory-enhanced approaches.

The use of theory-based nutrition education, the participation of husbands in the nutrition education intervention, the follow-up from early pregnancy through delivery, and the utilization of intervention methodologies are all strengths of this study. Further, the baseline characteristics across the three groups were comparable. However, the study was not without limitations. Participants were purposively assigned to the control or intervention groups. Due to the lack of randomization, there is a risk of bias. In addition, follow-up demographic characteristics were not collected during child delivery and many LBW risk factors such as the mother’s height and morbidity experience during pregnancy, which has previously been identified in studies, were not controlled for in this study. Further, all of the responses, with the exception of the MUAC and birth weight measurement, were relied on the women’s self-report, recall, and sincerity in responding to the questions.

## Conclusions

This study showed that a theory-based nutrition education intervention during pregnancy improved birth weight among pregnant women in Ethiopia. Pregnant women who received nutrition education with their husbands showed the greatest improvement. Dietary diversity was also found to be a mediator in the effect of the intervention on birth weight. This finding suggests that community-level education should be implemented through male engagement and theory-based techniques. The findings of this study can be used to draw key conclusions that will inform policies that seek to motivate males to support maternal and child nutrition. The result further provides a basis for further extensive research that will expand outcomes to include the long term sustenance of the behavior.

## Supporting information

S1 File Data collection tool. (DOCX)

S2 File Data. (SAV)

## Abbreviations

AIC: Akaike Information criterion
ANC: Antenatal care
ANOVA: Analysis of variance
CSA: Central Statistical Agency
DDS: Dietary Diversity Score
EDHS: Ethiopian Demographic Health Survey
GEE: Generalized Estimating Equation
HFIAS: Household Food Insecurity Assessment Scale
IRB: Institutional Review Board
LBW: Low birth weight
NIH: National Institutes of Health
PCA: Principal Component Analysis
PMTCT: Prevention of mother-to-child transmission
PTB: Pre-term birth
SPSS: Statistical Package for Social Science
TPB: Theory of Planned Behavior
WDDS: Women’s Dietary Diversity Score
WHO: World Health Organization
