# Supplementary material for: Effect of a theory-based nutrition education intervention during pregnancy through male partner involvement on newborns’ birth weights in Southwest Ethiopia. A three-arm community based Quasi-Experimental study
Source: PLoS One. 2023 Jan 17;18(1):e0280545. doi: 10.1371/journal.pone.0280545 (PMC9844912; doi:10.1371/journal.pone.0280545)
Supplement: S1 File — (DOCX) [file pone.0280545.s001.docx]

### Data collection tool English version

Date _________________

Code ________________

Instruction – Encircle the number which contains the correct answer or write the correct answer on the space provided.

**Part I: Socio-demographic Characteristics of the respondents**

| **S.N** | **Questions** | **Response** | **Code** | **Skip** |
| --- | --- | --- | --- | --- |
| **101** | How old are you? (in completed years) | ________________ |  |  |
| **102** | What is your current Marital status? | Married  Single  Divorced  Widowed | **1**  **2**  **3**  **4** |  |
| **103** | Number of family members | ___________________________ |  |  |
| **104** | Which Religion do you follow? | Orthodox  Protestant  Muslim  Catholic  Others (specify)____________ | **1**  **2**  **3**  **4**  **5** |  |
| **105** | To which ethinic group do you blong? | Oromo  Amhara  Guragie  Tigray  Others (specify)____________ | **1**  **2**  **3**  **4**  **5** |  |
| **106** | What is the highest education Level you ever attained? | Cannot read and write  Read and write  Elementary  Secondary and above | **1**  **2**  **3**  **4** |  |
| **107** | What is the highest education Level your husband ever attained? | Cannot read and write  Read and write  Elementary  Secondary and above | **1**  **2**  **3**  **4** |  |
| **109** | What is your Main occupation? | Farmer  House wife  Employee (GO/NGO)  Merchant  Student  Daily laboror  Others (Specify)_________ | **1**  **2**  **3**  **4**  **5**  **6**  **7** |  |
| **110** | What is the Main occupation of your husband? | Farmer  Employee (GO/NGO)  Merchant  Student  Daily laboror  Others (Specify)___ | 1  2  3  4  5  6 |  |

**Part II. Socio-economic characteristics**

| **S.N** | **Questions** | **Response** | **Coding** | **Skip** |
| --- | --- | --- | --- | --- |
| **201** | What is the main source of drinking water for members of your household?  *Circle ONLY ONE answer* | piped water  protected Dug well  unprotected dug well  protected spring  unprotected spring  rainwater  tanker truck  surface water (River, pond)  bottled water  others__________________ | 1  2  3  4  5  6  7  8  9  10 |  |
| **202** | Do you treat your water in any way to make it safer to drink? | Yes  No | 1  2 | If no go  to Q**204** |
| **203** | What do you usually do to the water to make it safer to drink?  *Circle ALL applicable answers* | boil  add bleach/chlorine  strain through a cloth  use water filter (ceramic/Sand/  let it stand and settle  others _______________ | 1  2  3  4  5  6 |  |
| **204** | What kind of toilet facility do members of your household usually use?  *Circle ONLY ONE answer* | flush or pour flush toilet  ventilated improved pit latrine (VIP)  pit latrine with slab  pit latrine without slab/ open pit  No facility/bush/field  others (specify _____________ | 1  2  3  4  5  6 |  |
| **205** | Do you share toilet facility with neibours? | Yes  No | 1  2 | If no go  to Q205 |
| **206** | How many families use the same toilet facility? | _____________________ |  |  |
| **207** | Does your household have:  *More than one answer is possible* | Electricity?  A watch?  A radio?  A television?  A mobile telephone  A non-mobile telephone?  A refrigerator?  A table?  A chair?  A bed?  An electric mitad? | 1  2  3  4  5  6  7  8  9  10  11 |  |
| **208** | What type of fuel does your household mainly use for cooking?  *Circle ONLY ONE answer* | electricity  biogas  kerosene  charcoal  wood  straw/shrubs/grass  animal dung  others (specify)____ | 1  2  3  4  5  6  7  8 |  |
| **209** | Do you have separate room which is used as kitchen? | Yes  No | 1  2 |  |
| **210** | Main material of the floor (observation)  *Circle ONLY ONE answer* | earth/ mud  wooden  ceramic tiles  cement/bricks  other [specify]_____________ | 1  2  3  4  5 |  |
| **211** | Main material of the roof (observation)  *Circle ONLY ONE answer* | thatch/leaf  plastic sheets  wood  corrugated iron sheet  cement  other [specify]______________ | 1  2  3  4  5  6 |  |
| **212** | Main material of the walls (observation)  *Circle ONLY ONE answer* | wooden and mud  wood/sticks  cement  stone with lime/cement  bricks  WOOD plank/SHINGLES other [specify]_________________ | 1  2  3  4  5  6  7 |  |
| **213** | What is now the primary source of income for this household?  *Circle ONLY ONE answer* | farming, including cash crops  livestock  employment/salary  petty trading (including sale fire- wood, charcoal, grass, localbrewery)  daily labor  handicrafts/artisan  remittances | 1  2  3  4  5  6  7 |  |
| **214** | Does this household own any land that can be used for agriculture? | Yes  No | 1  2 | If no go to Q**216** |
| **215** | If yes how much land does your household own? | ___________Hectars |  |  |
| **216** | Does this household own any livestock, herds, or farm animals? | Yes  No | 1  2 | If no stop |
| **217** | How many of the following animals do this household own?  **[PROBE AND MARK THAT ALL APPLY, MULTIPLE ANSWER IS POSSIBLE]** | cows  oxen, or bulls  calves  horses/donkeys/ mules  sheep and Goats  chickens | 1  2  3  4  5  6 |  |
| **218** | Does any member of the family have bank account? | Yes  No | 1  2 |  |
| **219** | Do you have home garden? | Yes  No | 1  2 |  |

**Part III. Information on Obstetric History**

| **S.N** | **Questions** | **Response** | **Code** | **Skip** |
| --- | --- | --- | --- | --- |
| **301** | Have you ever been pregnant before | Yes  No | 1  2 | If no go to Q**303** |
| **302** | If yes to question no 301, How many times | One  Two times  Three times  Four times and above | **1**  **2**  **3**  **4** |  |
| **303** | Have you ever had still birth | Yes  No  Don’t Know | 1  2  3 | If no or DK  go to Q**305** |
| **304** | If yes to question no 303, how many times? | _______________________ |  |  |
| **305** | Did you have any abortions? | Yes  No  Don’t Know | 1  2  3 | If no or DK go to Q**307** |
| **306** | If yes to question no 205, how many times? | _____________ |  |  |
| **307** | Have you attended ANC in your pregnancy? | Yes  No  Don’t Know | **1**  **2**  **3** |  |
| **308** | If yes to question no 307, how many times? | Once  Two times  Three times  Four times and above | **1**  **2**  **3**  **4** |  |
| **309** | At what gestational age did you start ANC attendance? | _______________ |  |  |

**Part IV: Dietary diversity score**

**Section One: 24 Hour Recall**

1. **Day of week that you are recalling.**

Monday Tuesday Wednesday

Thursday Friday Saturday

| Breakfast | Snack | Lunch | Snack | Dinner | Snack |
| --- | --- | --- | --- | --- | --- |
|  |  |  |  |  |  |

Sunday

1. **Women’s dietary diversity scores 9 groups of foods (1 item per food group type).**

**Dietary Diversity Questionnaire: Please describe the foods (meals and snacks) that you ate or drank yesterday during the day and night, whether at home or outside the home. Start with the first food or drink eaten in the morning.**

Write down all food and drinks mentioned. When composite dishes are mentioned, ask for the list of ingredients. When the respondent has finished, probe for meals and snacks not mentioned.

When the respondent recall is complete, fill in the food groups based on the information recorded above. For any food groups not mentioned, ask the respondent if a food item from this group was consumed.

**Section 2: Dietary Diversity**

**Please describe the foods (meals and snacks) that you ate yesterday during the day and night, whether at home or outside the home. Start with the first food eaten in the morning.**

| **Question number** | **Food group** | **Examples** | **Over 24 hrs**  **YES=1 NO=0** |
| --- | --- | --- | --- |
| 401 | CEREALS | corn/maize, teff, rice, wheat, barley, sorghum, millet or any other grains or foods made from these (e.g. bread, porridge or other grain products) *pasta, macaroni etc* |  |
| 402 | WHITE ROOTS AND TUBERS | white potatoes, godare, inset or other foods made from roots |  |
| 403 | VITAMIN A RICH VEGETABLES AND TUBERS | pumpkin, carrot, or sweet potato that are orange inside + *other locally available vitamin A rich vegetables (e.g. red sweet pepper)* |  |
| 404 | DARK GREEN LEAFY VEGETABLES | dark green/leafy vegetables, like cabbage pumpkin leaves |  |
| 405 | OTHER VEGETABLES | other vegetables (e.g. tomato, onion, green paper ) + *other locally available vegetables* |  |
| 406 | VITAMIN A RICH FRUITS | ripe mango, ripe papaya, and 100% fruit juice made from these + *other locally available vitamin A rich fruits* |  |
| 407 | OTHER FRUITS | other fruits, including wild fruits and 100% fruit juice made from these |  |
| 408 | ORGAN MEAT | liver, kidney, heart or other organ meats or blood-based foods |  |
| 409 | FLESH MEATS | beef, pork, lamb, goat, chicken |  |
| 410 | EGGS | eggs from chicken |  |
| 411 | FISH AND SEAFOOD | fresh or dried fish or shellfish |  |
| 412 | LEGUMES, NUTS AND SEEDS | Dried beans, dried peas Beans, lentils, nuts, seeds or foods made from these ( eg. shiro wet, kik wet, misir wet, shimbra kolo, bakela ashuk, adenguare, boloke) |  |
| 413 | MILK AND MILK PRODUCTS | milk, cheese, yogurt or other milk products |  |
| 414 | OILS AND FATS | oil, fats or butter added to food or used for cooking |  |
| 415 | SWEETS | sugar, honey or sweetened juice drinks, sugary foods such as chocolates, candies, cookies and cakes |  |
| 416 | SPICES, CONDIMENTS, BEVERAGES | spices (black pepper, salt), condiments (soy sauce, hot sauce), coffee, tea, alcoholic beverages |  |
| Individual level | Did you eat anything (meal or snack) OUTSIDE the home yesterday? | |  |

**Part V: Household food insecurity assessment scale**

| **No** | **Question** | **Response options** | **Code** |
| --- | --- | --- | --- |
| 501 | In the past four weeks, did you worry that your household would not have enough food? | 0 = No (skip to Q802)  1=Yes | ….\|___\| |
| 501A | If yes to above question, How often did this happen? | 1. Rarely (once or twice in the past four weeks) 2. Sometimes (three to ten times in the past four weeks) 3. Often (more than ten times in the past four weeks) | ….\|___\| |
| 502. | In the past four weeks, were you or any household member not able to eat the kinds of foods you preferred because of a lack of resources? | 0 = No (skip to Q803)  1=Yes | ….\|___\| |
| 502A | If yes to above question, How often did this happen? | 1. Rarely (once or twice in the past four weeks) 2. Sometimes (three to ten times in the past four weeks) 3. Often (more than ten times in the past four weeks) | ….\|___\| |
| 503 | In the past four weeks, did you or any household member have to eat a limited variety of foods due to a lack of resources? | 0 = No (skip to Q804)  1=Yes | ….\|___\| |
| 503A | If yes to above question, How often did this happen? | 1. Rarely (once or twice in the past four weeks) 2. Sometimes (three to ten times in the past four weeks) 3. Often (more than ten times in the past four weeks) | ….\|___\| |
| 504 | In the past four weeks, did you or any household member have to eat some foods that you really did not want to eat because of a lack of resources to obtain other types of food? | 0 = No (skip to Q805)  1=Yes | ….\|___\| |
| 504A | .If yes to above question, how often did this happen? | 1. Rarely (once or twice in the past four weeks) 2. Sometimes (three to ten times in the past four weeks) 3. Often (more than ten times in the past four weeks) | ….\|___\| |
| 505 | In the past four weeks, did you or any household member have to eat a smaller meal than you felt you needed because there was not enough food? | 0 = No (skip to Q806)  1=Yes | ….\|___\| |
| 505A | .If yes to above question, how often did this happen? | 1. Rarely (once or twice in the past four weeks) 2. Sometimes (three to ten times in the past four weeks) 3. Often (more than ten times in the past four weeks) | ….\|___\| |
| 506 | In the past four weeks, did you or any other household member have to eat fewer meals in a day because there was not enough food? | 0 = No (skip to Q807)  1=Yes | ….\|___\| |
| 506A | If yes to above question, how often did this happen? | 1. Rarely (once or twice in the past four weeks) 2. Sometimes (three to ten times in the past four weeks) 3. Often (more than ten times in the past four weeks) | ….\|___\| |
| 507 | In the past four weeks, was there ever no food to eat of any kind in your household because of lack of resources to get food? | 0 = No (skip to Q808)  1=Yes | ….\|___\| |
| 507A | If yes to above question, how often did this happen? | 1. Rarely (once or twice in the past four weeks) 2. Sometimes (three to ten times in the past four weeks) 3. Often (more than ten times in the past four weeks) | ….\|___\| |
| 508 | In the past four weeks, did you or any household member go to sleep at night hungry because there was not enough food? | 0 = No (skip to Q809)  1=Yes | ….\|___\| |
| 508A | If yes to above question, how often did this happen? | 1. Rarely (once or twice in the past four weeks) 2. Sometimes (three to ten times in the past four weeks) 3. Often (more than ten times in the past four weeks) | ….\|___\| |
| 509 | In the past four weeks, did you or any household member go a whole day and night without eating anything because there was not enough food? | 0 = No (Last)  1=Yes | ….\|___\| |
| 509A | If yes to above question, how often did this happen? | 1. Rarely (once or twice in the past four weeks) 2. Sometimes (three to ten times in the past four weeks) 3. Often (more than ten times in the past four weeks) | ….\|___\| |

**Part VI: Anthropometric MEASUREMENT RESULTS**

| **S. no** | **Measurement** | **Result** |
| --- | --- | --- |
| 601 | MUAC in cms | _______________________ |
| 602 | **Sex of the child** | 1. **Male** 2. **Female** |
| 603 | **Birth weight (in gram)** | **_________________________** |

**Thank you!**

**QUESTIONAIRE AFAN OROMO VERSION**

**Kutaa I: Haala hawwaasummaa kan ilaalu**

| **Lakk.** | **Gaaffilee** | **Deebii** | **Koodii** | **Irradarbi** |
| --- | --- | --- | --- | --- |
| **101** | Umuriin kee meeqa? (waggaa guutuudhaan) | ________________ |  |  |
| **102** | Haala gaa’elaa kee yaroo ammaa? | Hinheerumne  Kanhinheerumne  Kanhiikte  Kan abbaan manaa irraa du’e | **1**  **2**  **3**  **4** |  |
| **103** | Baayyini maatii keetii meeqa? |  |  |  |
| **104** | Amantii kam hordofta? | Ortodoksii  Piroteestaantii  Musliima  Kaatoolikii  Kan biro(Ibsi)____________ | **1**  **2**  **3**  **4**  **5** |  |
| **105** | Sab-lammiin kee kam? | Oromoo  Amaara  Guraagee  Tigiree  Kanbiroo(Ibsi)__________ | **1**  **2**  **3**  **4**  **5** |  |
| **106** | Sadarkaa barnootaa hagam hordofteetta? | hinbaranne  Barreessuuf dubbisuu qofa  Sadarkaa 1ffaa (1-8)  Sadarkaa lammaffaa fi isaa ol | **1**  **2**  **3**  **4** |  |
| **107** | Abbaan manaakee Sadarkaa barnootaa hagamitti hordofeera? | hinbaranne  Barreessuuf dubbisuu qofa  Sadarkaa 1ffaa (1-8)  Sadarkaa lammaffaa fi isaa ol | **1**  **2**  **3**  **4** |  |
| **109** | Hojiin kee yaroo ammaa maal? | Qacaramtuu (GO/NGO)  Haadha warraa  Daldaltuu  Barattuu  Qonnaan bultuu  Hojii guyyaa  Kanbiroo (Ibsi)_________ | **1**  **2**  **3**  **4**  **5**  **6**  **7** |  |
| **110** | Hojii abbaa warraa keetii yeroo ammaa kana maal? | Qacaramaa (GO/NGO)  Daldalaa  Barataa  Qonnaan bulaa  Hojii guyyaa  Kanbiroo (Ibsi)_________ | **1**  **2**  **3**  **4**  **5**  **6** |  |

**Kutaa II: Haala dinagdee kan ilaalu**

| **Lakk.** | **Gaaffilee** | **Deebii** | **Koodii** | | **Irradarbi** |
| --- | --- | --- | --- | --- | --- |
| **201** | Maddi bishaan dhugaatii maatii keessanii maali?  (Deebiitokkoqofafiladhu.) | Bishaan boombaa  Bishaan boollaa kunuunfame  Bishaan boollaa kan hinkunuunfamne  Burqituu gabbate  Burqituu hingabbatin  Bishaan bokkaa  Bishaan qodaatti kuufame  Bishaanyaa’aa/walittiqabame  Bishaan ashagamaa  Kanbiroo__________________ | 1  2  3  4  5  6  7  8  9  10 | |  |
| **202** | Bishaan dhugaatii keessan karaa adda addaa nikunuunsituu? | Eeyyee  Lakki | 1  2 | | 2 204 |
| **203** | Bishaan calaluuf mala akkamii fayyadamtu?  (Deebii tokkoo ol deebisuun nidanda’ama.) | Danfisuu  Kilooriini iitti naquu  Uffataan calaluu  Fiilterii fayyadamuu  Yeroo dheeraa turee akka calalu  Kanbiroo _______________ | 1  2  3  4  5  6 | |  |
| **204** | Maatiin keessan mana fincaanii akkamii fayyadamu?  (Deebii tokko qofa filadhu.) | Bishaaniin kan deemu  VIP  Mana fincaanii boollaa  Boolla duwwaa  Bakkeetti bobba’uu  Kanbiroo (Ibsi) _____________ | 1  2  3  4  5  6 | |  |
| **205** | Mana fincaanii matii olla waliin nifayadamtuu? | Eyyee  Lakkii | 1  2 | | 2 207 |
| **206** | Matii meeqatu mana fincaanii tokkotti fayyadama? | ____________________ |  | |  |
| **207** | Maatin kessaan wantoota tarreeffaman kana qabuu? |  | Yes | No |  |
|  |  | Elektirisiitii? | 1 | 2 |  |
|  |  | Sa’atii? | 1 | 2 |  |
|  |  | Raadiyoo? | 1 | 2 |  |
|  |  | Televiiziyoonii? | 1 | 2 |  |
|  |  | Bilbila moobaayilaa? | 1 | 2 |  |
|  |  | Bilbila moobaayila hintaane? | 1 | 2 |  |
|  |  | Refirijireetarii? | 1 | 2 |  |
|  |  | Xarabbeezzaa? | 1 | 2 |  |
|  |  | Barcuma? | 1 | 2 |  |
|  |  | Siree? | 1 | 2 |  |
|  |  | Eelee elektirikii? | 1 | 2 |  |
| **208** | Nyaata bilcheessuuf maalitti fayyadamtu?  (Deebii tokko qofa filadhu.) | Elektirisiitii  Baayoogaasii  Gaasiiadii  Kasala  Qoraan  straw/shrubs/grass  animal dung  Kanbiroo (Ibsi)___________ | 1  2  3  4  5  6  7  8 | |  |
| **209** | Kutaa itti nyaata bilcheeffattan qofaatti qabduu? | Eeyyee  Lakki | 1  2 | |  |
| **210** | Manni jireenyaa keessan lafti isaa maal irraa hojjetame? (Ni ilaalama)  (Deebii tokko qofa filadhu.) | Biyyoo  Mukarraa  Seeraamikii  Simintooirraa  Kanbiroo(Ibsi)_____________ | 1  2  3  4  5 | |  |
| **211** | Manni jireenyaa keessan xaaraan isaa maal irraa hojjetame? (Ni ilaalama)  (Deebii tokko qofa filadhu.) | Citaa  Pilaastikii  Muka  Qorqorroo  Kanbiroo(Ibsi)______________ | 1  2  3  4  5 | |  |
| **212** | Manni jireenyaa keessan keenyann iisaa maal irraa hojjetame? (Ni ilaalama)  (Deebii tokko qofa filadhu.) | Mukaa fi biyyoo  Muka irraa  Simintoo  Dhagaa fi simintoo  Biriikii  Kanbiroo (Ibsi)_____________ | 1  2  3  4  5  6 | |  |
| **213** | Yeroo ammaa maddi galii maatii keessanii maali?  (Deebii tokko qofa filadhu.) | Qonnaa  Horii horsiisuu  Qacarrii/Miindaa  Daldala (Qoraan, kasala, dhugaatii aadaa…..)  Hojii guyyaa  Hojii harkaa  Qarshii nama biraa irraa kan argatan | 1  2  3  4  5  6  7 | |  |
| **214** | Maatiin keessan lafa qonnaaf oolu qabaa? | Eeyyee  Lakki | 1  2 | | 2 216 |
| **215** | Yoo Eeyyee jette, maatiin keessan lafa hammamii qaba? (Hectaaraan) | _________________ |  | |  |
| **216** | Maatiin keessan horii manaa qabduu? | Eeyyee  Lakki | 1  2 | | Yoo lakki jette dhaabi. |
| **217** | Maatiin keessan horii armaan gadii keessaa kanneen kam qabdu?  **(Kan ilaallatu hunda guuti, Deebii tokkoo ol deebisuun ni danda’ama.)** | Sa’a annanii  Sangaa  Jabbii  Farda/Harree/Gaangee  Hoolaa  re’ee  Lukkuu | 1  2  3  4  5  6 | |  |
| **218** | Maatii kessan keessaa namni baankiitti ykn waldaa liqii fi qusannootti qusatu jira? | Eyyee  Lakkii | 1  2 | |  |
| **219** | Qonnaa goroo (Home garden) qabduu | Eyyee  Lakkii | 1  2 | |  |

**Kutaa III. Haala da’umsaa fi isaan kan walqabatu**

| **Lakk.** | **Gaaffilee** | **Deebii** | **Koodii** | **Irradarbi** |
| --- | --- | --- | --- | --- |
| **301** | Kana dura ulfooftee beektaa? | Eeyyee  Lakki | 1  2 | Yoo lakki jette gara Gaaffii **303** dhaqi |
| **302** | Gaaffii **301** yoo Eeyyee jettee deebiste yeroo meeqa ulfoofte? | Yeroo tokko  Yeroo lama  Yeroo sadii  Yerooa furii fi isaa ol | **1**  **2**  **3**  **4** |  |
| **303** | Kanaan dura mucaa du’aa deessee beektaa? | Eeyyee  Lakki | 1  2 | Yoo hinbeeku jette gara Gaaffii **305**dhaqi |
| **304** | Gaaffii **303** yoo Eeyyee jettee deebiste yeroo meeqa? | ______________ |  |  |
| **305** | Ulfi sirraa ba’ee beekaa? | Eeyyee  Lakki | 1  2 | Yoo hinbeeku jette gara Gaaffii **307**dhaqi |
| **306** | Gaaffii **305** yoo Eeyyee jettee deebiste yeroo meeqa? | _____________ |  |  |
| **307** | Hordoffii yeroo ulfaa ulfa isa kana duraa iratti hordoftee beektaa? | Eeyyee  Lakki | **1**  **2** |  |
| **308** | Gaaffii **307** yoo Eeyyee jettee deebiste yeroo meeqa? | Yeroo tokko  Yeroo lama  Yeroo sadii  Yeroo afurii fi isaaol | **1**  **2**  **3**  **4** |  |
| **309** | Ulfa ji’a meeqaa taatee hordoffii jalqabde? | __________ |  |  |

**Kutaa V: Haala nyaataa Gosa garaa garaa**

**Guyyaa torbanii .**

Wixata Kipxata Robii

Kamisa Jimaata Sanbata duraa

Dilbata

**Nyaata mana Keessatti nyaatamu kan gosa 9 qabatu**

Nyaata kaleessa galgala, guyyaa manatti ykn alatti nyaattee tarreessi .Nyaata jalqaba ganama nyaatte ykn dhugde waliin eegali.

Namoonni gaafataman gaaffii kana gubbaa jiiru erga xumranii boodaa nyaata caqasaman gosa gosan gutaa. Nyaata gosa kamiiyyuu kan hin caqasamiin jiruu taanaan, nyaataa gosa kana fayyadamuu isaanii gaafachu.

| L/G | Gosa Nyaataa | Fakkeenya | **Sa’atii 24 kessatti**  **1=Eeyee 2=Miti** |
| --- | --- | --- | --- |
| 401 | Midhaan ykn kan firiin isaanii  nyaatamu | Boqolloo ,xaafii, Ruuzii , Qamadii , bisingaa/Boobee , daaguzaa ykn nyaata  kanneen warra kana irraa hojjataman ,buddeena, daabboo, marqaa ,pasta fi kan biroo |  |
| 402 | Hundee adii | Dinnicha adii kaasaavaa adii fi kanneen biroo |  |
| 403 | Muduralee Viitammin A dhaan badhaadhoo ta’an | Buqqee, Kaarotii , cemcemee , mosee fi kanneen biroo |  |
| 404 | Muduralee magariisa ta.an | raafuu, baala buqqee, ,muduraalee magariisa nannootti biqilan |  |
| 405 | Kuduraalee kan biroo | Timaatimii, shunkurtii, mimixa qaaraa |  |
| 406 | Kuduralee Viitammin A dhaan badhaadhoo ta’an | Pappayaa, mangoo, fi cuunfaa isaan kana irraa hojjetaman |  |
| 407 | Kuduraalee kan biroo | Ija mukaa garaa geraa bosona keessatti kan argaman |  |
| 408 | Foon kale fi tiruu | Tiruu, kalee, Onnee fi kan biroo |  |
| 409 | Foon dimina | Kan saawwaa, kan boyee kan jabbii,reettii, Indaaqoo,daakkiiyee fi kan biro |  |
| 410 | Anqaaquu | Anqaaquu indaaqqoo |  |
| 411 | Qurxummii | Qurxummii |  |
| 412 | Akuletarii Nuugii fi sanyiin isaanii kan nyaatamu | Baaqelaa,atarii, misira, saalixii , nuugii fi kan biro isann irraa kan hojjetaman kan akka shiroo,Kikkii |  |
| 413 | Aannanii fi bu’aa aannanii | Aannaan , baaduu ,itittuu ykn kan biroo |  |
| 414 | Coomaa fi Zayita | Zayita,coomaa fi dhadhaa |  |
| 415 | Nyaata mimminyaa’aa | Dammaa, sukkaara, cuunfaa waantota sukkaara of keessaa qabaniin hojjetaman, chokkolaataa,karameellaa,keekii garaagaraa |  |
| 416 | Qimamii garaagaraa fi dhugaatii lallaafaa | Mixmixa, ashaboo fi buna shaayee, dhugaatii lallaafaa |  |
|  | Kaleessa nyaata ykn dhugaatii alaa fayyadamte qabdaa? | |  |

**Kutaa V: Gaaffilee haala wabii nyaataa maatii qorachuuf qophaa’an**

| L/G | Gaaffii | Deebii (tokko filadhu) | | | koodii |
| --- | --- | --- | --- | --- | --- |
| 501 | Torban afran darban keessatti maatiin keessan nyaata gahaa ta’e hin qabu jechuun yaaddoftanii turtanii? | | 0=Lakki (gara 502)  1=Eeyyee | | …/___/ |
| 501.a | Yaaddoon armaan olii kun al meeqa isin mudatee? | 1=Yeroo muraasa (baatii darbeetti al 1-2tti)  2=Altokko tokko (baatii darbeetti al 3-10tti)  3=Yeroo hedduu (baatii darbeetti yeroo 10 ni olii) | | | …/___/ |
| 502. | Torban afran darban keessatti, isin ykn miseensi maatii keessanii qarshii ykn qabeenya dhabuun gosa nyaataa filattan osoo hin nyaatin haftanii jirtuu? | | | 0=Lakki (gara 503)  1=Eeyyee | …/___/ |
| 502.a | Rakkoon armaan olii kun al meeqa isin mudatee? | 1=Yeroo muraasa (baatii darbeetti al 1-2tti)  2=Altokko tokko (baatii darbeetti al 3-10tti)  3=Yeroo hedduu (baatii darbeetti yeroo 10 ni olii) | | | …/___/ |
| 503. | Torban afran darban keessatti, isin ykn miseensi maatii keessanii qarshii yhn qabeenyaa dhabuun gosa nyaataa muraasa qofa nyaachuuf yeroon itti dirqamtan jiraa? | | | 0=Lakki (gara 504)  1=Eeyyee | …/___/ |
| 503.a | Rakkoon armaan olii kun al meeqa isin mudatee? | 1=Yeroo muraasa (baatii darbeetti al 1-2tti)  2=Altokko tokko (baatii darbeetti al 3-10tti)  3=Yeroo hedduu (baatii darbeetti yeroo 10 ni olii) | | | …/___/ |
| 504. | Torban afran darban keessatti, isini ykn miseensi maatii keessanii qarshii ykn qabeenyaa dhabuun akaakuu nyaataa nyaachuu hin feenee nyaachuuf yeroon itti dirqamtan jira turee? | | | 0=Lakki (gara 505)  1=Eeyyee | …/___/ |
| 504.a | Rakkoon armaan olii kun al meeqa isin mudatee? | 1=Yeroo muraasa (baatii darbeetti al 1-2tti)  2=Altokko tokko (baatii darbeetti al 3-10tti)  3=Yeroo hedduu (baatii darbeetti yeroo 10 ni olii) | | | …/___/ |
| 505. | Torban afran darban keessatti, isin ykn miseensi maatii keessanii nyaanni gahaan waan hin jirreef nyaata hanga soorachuu barbaaddaniin gadiitti soorachuuf yeroon itti dirqamtan jira turee? | | | 0=Lakki (gara 506 )  1=Eeyyee | …/___/ |
| 505.a | Rakkoon armaan olii kun al meeqa isin mudatee? | 1=Yeroo muraasa (baatii darbeetti al 1-2tti)  2=Altokko tokko (baatii darbeetti al 3-10tti)  3=Yeroo hedduu (baatii darbeetti yeroo 10 ni olii) | | | …/___/ |
| 506. | Torban afran darban keessatti, isin ykn miseensi maatii keessanii nyaanni gahaan waan hin jirreef guyyaatti yeroo muraasaa qofa soorachuuf yeroon itti dirqamtan jira turee? | | | 0=Lakki (gara 507)  1=Eeyyee | …/___/ |
| 506.a | Rakkoon armaan olii kun al meeqa isin mudatee? | 1=Yeroo muraasa (baatii darbeetti al 1-2tti)  2=Altokko tokko (baatii darbeetti al 3-10tti)  3=Yeroo hedduu (baatii darbeetti yeroo 10 ni olii) | | | …/___/ |
| 507. | Torban afran darban keessatti, qarshiin ykn qabeenyi waan hi jirreef nyaata akaakuu kamiiyyuu mana keessaa yeroon itti dhabdan ni jira turee? | | | 0=Lakki (gara 508 )  1= Eeyyee | …/___/ |
| 507.a | Rakkoon armaan olii kun al meeqa isin mudatee? | 1=Yeroo muraasa (baatii darbeetti al 1-2tti)  2=Altokko tokko (baatii darbeetti al 3-10tti)  3=Yeroo hedduu (baatii darbeetti yeroo 10 ni olii) | | | …/___/ |
| 508. | Torban afran darban keessatti galgala galgala isin ykn miseensi maatii keessanii nyaatni gahaan waan hin jirreef beela’aa ykn garaa duwwaatti ciisuuf yeroon itti dirqamtan jira turee? | | | 0=Lakki (gara 509)  1= Eeyyee | …/___/ |
| 508.a | Rakkoon armaan olii kun al meeqa isin mudatee? | 1=Yeroo muraasa (baatii darbeetti al 1-2tti)  2=Altokko tokko (baatii darbeetti al 3-10tti)  3=Yeroo hedduu (baatii darbeetti yeroo 10 ni olii) | | | …/___/ |
| 509 | Torban afran darban keessatti, isin ykn miseensi maatii keessanii nyaatni gahaan waan hin jirreef galgalaa fi guyyaa guutuu beela’aa ykn garaa duwwaa yeroon itti turuuf dirqamtan jira turee? Al meeqaa? | | | 0=Lakki ( last)  1=Eeyyee | …/___/ |

**Section –VI: Anthropometric data**

|  | Characteristics | Response category |
| --- | --- | --- |
| 601 | MUAC in cm |  |
| 602 | Haala daa’ima dhalatee (Birth Outcome) | 1. Mucaa fayyaa 2. Mucaa du’aa |
| 603 | Saala mucaa | 1. Dhiira 2. Dhala |
| 604 | Ulfaatinna qaamaa mucaa (graamaan) | ______________________ |

**Galatoomaa!**
